# Supplementary material for: Wild Boar Attacks on Hunting Dogs in Czechia: The Length of the Hunting Season Matters
Source: Animals (Basel). 2025 Jan 8;15(2):130. doi: 10.3390/ani15020130 (PMC11758641; doi:10.3390/ani15020130)
Supplement: Supplementary file 1 [file animals-15-00130-s001.zip › animals-3405516-supplementary.pdf]

Supplementary Table S1 Comprehensive questionnaire: The table presents a comprehensive list of questions included in our questionnaire. The questionnaire was structured to cover topics such as the characteristics of the hunting grounds, the organization and frequency of collective hunts, the number of participants and dogs, and incidents involving wild boar attacks.

**INFORMED CONSENT STATEMENT**	
	In accordance with applicable GDPR regulations, I consent to the processing and publication of data related to hunting grounds as part of this study.

	**THE HUNTING GROUND**	
1.	**In which cadastral area is your hunting ground located?**	
		Fill in the name of the hunting ground
2.	**How large is your hunting ground?**	
		Enter the area in hectares.
	**How many hectares of it are forest?**	
		Fill in the number of hectares.
3.	**How many collective hunts did you organize in a hunting season (from 1.4. of the current year until 31.3. next year)?** Fill in the number of drive hunts in each hunting season.	
		2012
		2013
		2014
		2015
		2016
4.	**When do you usually go on your first collective hunt in the hunting season?**	
		The name of the month.
5.	**When is your last collective hunt in the hunting season?**	
		The name of the month.
6.	**What are the time intervals between collective hunts?**	
		Number of days between collective hunts.
7.	**On average, how many people participate in a collective hunt?**	
		Number of persons.
8.	**How many of these participants are beaters?**	
		The number of beeters.
9.	**How many wild boars do you shoot in a collective hunt per hunting season?** Add the number of pieces per month in hunting season.	

**season 1**	X.12	**s. 3**	XII.14
	XI.12		I.15
	XII.12	**season 4**	X.15
	I.13		XI.15
**season 2**	X.13		XII.15
	XI.13		I.16
	XII.13	**season 5**	X.16
	I.14		XI.16
**s. 3**	X.14		XII.16
	XI.14		I.17

10.	**How many dogs do you have on average in a collective hunt?**	

	0-5
	6-10
	≤10
**WILD BOAR ATTACKS**	

11. **How many attacks on dogs do you experience in a collective hunt per hunting season?**

	2012
	2013
	2014
	2015
	2016

12. **Please select the month/months in the relevant hunting season and state the number of MILD dog injuries:**

**season 1**	X.12	**s. 3**	XII.14
	XI.12		I.15
	XII.12	**season 4**	X.15
	I.13		XI.15
**season 2**	X.13		XII.15
	XI.13		I.16
	XII.13	**season 5**	X.16
	I.14		XI.16
**s. 3**	X.14		XII.16
	XI.14		I.17

13. **Please select the month/months in the relevant hunting season and state the number of SEVERE dog injuries:**

**season 1**	X.12	**s. 3**	XII.14
	XI.12		I.15
	XII.12	**season 4**	X.15
	I.13		XI.15
**season 2**	X.13		XII.15
	XI.13		I.16
	XII.13	**season 5**	X.16
	I.14		XI.16
**s. 3**	X.14		XII.16
	XI.14		I.17

14. **Please select the month/months in the relevant hunting season and state the number of FATAL dog injuries:**

**season 1**	X.12	**s. 3**	XII.14
	XI.12		I.15
	XII.12	**season 4**	X.15
	I.13		XI.15
**season 2**	X.13		XII.15
	XI.13		I.16
	XII.13	**season 5**	X.16
	I.14		XI.16
**s. 3**	X.14		XII.16
	XI.14		I.17

Supplementary Table S2 A set of *a priori* hypotheses.

The length of season (hunting season)
The length of season (hunting season) the interval between driven hunts
The length of season (hunting season) the length of season interval between driven hunts
Number of dogs (hunting season)
Month
The length of season * number of driven hunts (hunting season) the interval between driven hunts
Number of beaters per driven hunt (hunting season)
Forest area of hunting ground
The interval between driven hunts
Number of hunters per driven hunt (hunting season)
The length of season * number of driven hunts (hunting season)
Number of wild boars harvested in a hunting ground (hunting season)
Hunting season
Area of hunting ground
Number of participants (hunting season)
Number of driven hunts (hunting season)
The length of season
Null model

Supplementary Table S3 Five best candidate models ranked by the five best fit criteria and the null model.

Model	AIC	AICC	BIC	CAIC	HQIC	Rank AIC	Rank _AIC C	Rank _BIC	Rank _CAI C	Rank _HQI C
The length of season	416.81	416.88	420.14	422.14	418.01	1	1	1	1	1
The length of season (hunting season)	423.90	423.96	427.23	429.23	425.09	2	2	2	2	2
The length of season (hunting season), the interval between driven hunts	429.73	429.80	433.06	435.06	430.93	3.5	3.5	3.5	3.5	3.5
The length of season (hunting season), the length of season interval between driven hunts	429.73	429.80	433.06	435.06	430.93	3.5	3.5	3.5	3.5	3.5
Number of dogs (hunting season)	432.81	432.87	436.14	438.14	434.00	5	5	5	5	5
Null Model	548.70	548.72	551.97	552.97	550.02	6	6	6	6	6

Supplementary Table S4 Comparison of the best model to the null model.

Variable	Null_Model	Best_model	Δ	Relative_information_loss
AIC	548.70	416.81	131.88	2.30E-29
AICC	548.72	416.88	131.84	2.35E-29
BIC	551.97	420.14	131.83	2.37E-29
CAIC	552.97	422.14	130.83	3.91E-29
HQIC	550.02	418.01	132.01	2.16E-29

Supplementary Table S5 Estimates, standard error, and 95% confidence interval for best fitting GLMM model for the log-transformed number of attacks.

Effect	Estimate	StdErr	Lower	Upper
Intercept	-1,56	0,36	-2,30	-0,82
The length of season	0,83	0,13	0,57	1,09
